# FOXM1c promotes oesophageal cancer metastasis by transcriptionally regulating IRF1 expression

**DOI:** 10.1111/cpr.12553

**Published:** 2018-11-28

**Authors:** Yuzhen Zhou, Qi Wang, Li Chu, Weixing Dai, Xiaozhou Zhang, Jianfeng Chen, Long Zhang, Peipei Ding, Xin Zhang, Hongyu Gu, Pingzhao Zhang, Ling Li, Wei Zhang, Luying Li, Xinyue Lv, Danlei Zhou, Guoxiang Cai, Liang Chen, Kuaile Zhao, Weiguo Hu

**Affiliations:** ^1^ Fudan University Shanghai Cancer Center Institutes of Biomedical Sciences, Shanghai Medical College Fudan University Shanghai China; ^2^ Department of Radiation Oncology, Fudan University Shanghai Cancer Center, Shanghai Medical College Fudan University Shanghai China; ^3^ Department of Colorectal Surgery, Shanghai Medical College Fudan University Shanghai China; ^4^ Key Laboratory of Functional Protein Research of Guangdong Higher Education, Institute of Life and Health Engineering, College of Life Science and Technology Jinan University Guangzhou China

## Abstract

**Objectives:**

We aimed to elucidate the role and molecular mechanisms of FOXM1 in regulating metastasis in oesophageal squamous cell carcinoma (ESCC) as well as its clinical implications.

**Materials and methods:**

The expression levels of four isoforms of FOXM1 were analysed by real‐time PCR. Next, genetically modification using overexpression and RNAi systems and transwell were employed to examine FOXM1c function in invasion and migration. Dual luciferase and ChIP assays were performed to decipher the underlying mechanism for transcriptional regulation. The expression levels of FOXM1 and IRF1 were determined by immunohistochemistry staining in ESCC specimens.

**Results:**

The FOXM1c was predominantly overexpressed in ESCC cell lines compared to the other FOXM1 isoforms. Ectopic expression of FOXM1c promoted invasion and migration of ESCC cells lines, whereas downregulation of FOXM1c inhibited these processes. Moreover, FOXM1c expression was positively correlated with IRF1 expression in ESCC cell lines and tumour specimens. IRF1 is, at least in part, responsible for FOXM1c‐mediated invasion and migration. Mechanistically, we identified IRF1 as a transcriptional target of FOXM1c and found a FOXM1c‐binding site in the IRF1 promoter region. Furthermore, high expression levels of both FOXM1c and IRF1 were positively associated with low survival rate and predicted a poor prognosis of oesophageal cancer patients.

**Conclusion:**

FOXM1c promotes the metastasis by transcriptionally targeting IRF1 and may serve as a potential prognostic predictor for oesophageal cancer.

## 1 INTRODUCTION

The incidence of oesophageal cancer has rapidly increased in the United States and western countries over the past decades.^1^, ^2^ In Asia, oesophageal cancer is also one of the most aggressive cancers, with a high prevalence.^3^ As a major subtype of oesophageal cancer, oesophageal squamous cell carcinoma (ESCC) accounts for approximately 90% of oesophageal cancers and has been ranked as the fourth leading cause of cancer‐related mortality in China.^4^ Oesophageal cancer has a poor prognosis due to early metastasis and direct invasion, the 5‐year overall survival rate of which is less than 20%.^5^ The majorities of oesophageal cancer patients develop resistance to both chemo‐ and radiotherapy despite initial response.^6^, ^7^, ^8^, ^9^, ^10^ Moreover, patients with resistance to these treatments are frequently diagnosed with metastasis.^11^, ^12^ Therefore, elucidation of the mechanisms involved in oesophageal cancer metastasis is urgently needed.

FOXM1 is strongly overexpressed in almost all types of human cancers and is highly associated with cancer progression, including that of oesophageal cancer.^13^ In general, there are four distinct isoforms of FOXM1: FOXM1a, FOXM1b, FOXM1c and FOXM1d, due to the alternative splicing of its exons Va and VIIa.^13^, ^14^ FOXM1a, which retains both exons Va and VIIa, is predominantly located in the cytoplasm and is transcriptionally inactive, probably due to the disruption of its transactivation domain.^15^ Both FOXM1b (without both Va and VIIa) and FOXM1c (with only exon Va), which are predominantly located in the nucleus, play similar but not identical biological roles and have different binding affinities and partners.^13^ We recently identified FOXM1d as a novel FOXM1 isoform that has exon VIIa but lacks exon Va and is predominantly located in the cytoplasm; thus, it does not have direct transcription‐regulating functions.^14^ FOXM1 has widely been recognized as a proliferation‐specific oncogenic transcription factor^13^ that transcriptionally regulates a number of genes that are involved in the G2‐M progression, such as Plk1, AuroraB, Cyclin B1, CDC25B, CENP‐A and Survivin.^16^, ^17^ In addition, FOXM1 plays an essential role in the regulation of a wide spectrum of biological processes, such as inflammation, metabolism, angiogenesis, apoptosis and metastasis.^18^, ^19^, ^20^, ^21^ Overexpression of FOXM1 is highly associated with tumour cell survival, epithelial‐to‐mesenchymal transition (EMT), chemo‐/radio‐resistance and metastasis.^18^, ^19^ Downregulation of FOXM1 inhibits matrix metalloproteinases (MMPs), including MMP2 and MMP9, and inhibits nasopharyngeal carcinoma metastasis.^22^, ^23^ FOXM1 is associated with metastasis in colorectal cancer through induction of EMT.^24^ In oesophageal cancer, FOXM1 expression positively correlates with poor prognosis,^25^ and miR‐204 regulates the invasion and EMT by direct targeting the 3'UTR of FOXM1.^26^

Although the isoforms showed obviously different intracellular distributions and action mechanisms, there are few reports investigating the distinct isoforms of FOXM1 in promoting cancer metastasis, a critical step for late‐stage progression. FOXM1b could be SUMOylated at lysine 463, thus promoting breast cancer metastasis.^27^ FOXM1c, which is predominantly overexpressed in pancreatic cancer, transcriptionally upregulated urokinase‐type plasminogen activator receptor (uPAR), thus contributing to pancreatic cancer metastasis.^28^ FOXM1d interacted with and further activated ROCKs, promoting colorectal cancer EMT and metastasis.^14^ However, few studies have specifically investigated the distribution, abundance and roles of individual FOXM1 isoforms in oesophageal cancer metastasis.

In this study, we investigated the abundance of each FOXM1 isoform in oesophageal cancer cells and found that FOXM1c was the predominant isoform. FOXM1c modulates oesophageal cancer invasion and migration by regulating IRF1 transcription and subsequently MMP2/9 expression. We further observed that both FOXM1c and IRF1 were positively correlated with poor prognosis and low survival rate in oesophageal cancer patients. These findings suggest that FOXM1c and IRF1 may be potential diagnostic biomarkers and drug targets for oesophageal cancer.

## 2 MATERIALS AND METHODS

### 2.1 Cell culture

Four human ESCC cell lines, KYSE510, KYSE180, Eca109 and TE1, were cultured in plastic flasks as adherent monolayers in RPMI‐1640 medium (HyClone, South Logan, UT) supplemented with 10% foetal bovine serum (Gibco, Thermo Fisher Scientific, Waltham, MA) and 5% penicillin‐streptomycin antibiotics (Gibco, Thermo Fisher Scientific) and were maintained at 37°C in a humidified incubator with 5% CO_2_.

### 2.2 Plasmids, siRNA and transfection

For generation of the overexpression vector of FOXM1c and RNAi vectors of FOXM1c and IRF1, the coding sequence (CDS) and the shRNA sequences of FOXM1c and IRF1 were inserted into the pCDH‐puro and pLKO.1 vectors (Promega, Madison, WI) and were packaged into lentiviruses. The sequences of the shRNAs of FOXM1c and IRF1 are as follows: sh‐FOXM1c‐1#: 5'‐CCGGGGACCCAGGGTCTCCACAATTCTCGAGAATTGTGGAGACCCTGGGTCCTTTTTG‐3', sh‐FOXM1c‐2#: CCGGATTGCCCGAGCACTTGGAATCCTCGAGGATTCCAAGTGCTCGGGCAATTTTTTG. sh‐IRF1‐1#: 5'‐CCGGGGCTAGAGATGCAGATTAATTCTCGAGAATTAATCTGCATCTCTAGCCTTTTTG‐3', sh‐IRF1‐2#: 5'‐CCGGGGGCTCATCTGGATTAATAAACTCGAGTTTATTAATCCAGATGAGCCCTTTTTG‐3'. Two restoring plasmids with synonymous mutation of FOXM1c in shRNA targets region were designed and constructed using KOD‐Plus‐Mutagenesis kit (TOYOBO, Osaka, Japan). pCDH‐puro‐FOXM1c was used as PCR template. Mutation primers were listed as follows: shFOXM1c‐1#‐Restore‐F: 5'‐GCTTCCCGAGCACTTGGAATCACAG‐3', shFOXM1c‐1#‐Restore‐R: 5'‐TGTGGAGACCCTGGGTCCAGTGGCT‐3'; shFOXM1c‐2#‐Restore‐F: 5'‐TTTAGAGTCACAGCAGAAACGACCG‐3', shFOXM1c‐2#‐Restore‐R: 5'‐ TGCTCGGGCAATTGTGGAGACCCTG‐3'. Stable cell lines were established by screening with puromycin at a concentration of 5 μg/mL 48 hours after infection with the lentivirus and confirmed by Western blot analysis. The cells were seeded into six‐well plates in antibiotic‐free medium at 50% density and transfected with the FOXM1c siRNA duplex (1#: 5'‐CCCAGGGUCUCCACAAUUG‐3'; 2#: AUUGCCCGAGCACUUGGAAUC), IRF1 siRNA duplex (1#: 5'‐CCAACUUUCGCUGUGCCAU‐3', 2#: 5'‐CCAGAUCCCAUGGAAGCAU‐3') or control siRNA duplex (5'‐UUCUCCGAACGUGUCACGU‐3') oligonucleotides at a final concentration of 20 μmol/L using Lipofectamine 3000 (Life Technology, Gaithersburg, MD) according to the manufacturer's instructions. After 48 hours, cells were collected for qPCR, Western blot and transwell assays. For FOXM1c rescue assay, the restoring plasmids were transiently transfected into sh*FOXM1c*‐1# and sh*FOXM1c*‐2# Eca109 stable cell lines with 500 ng per well in six‐well plate. RNA and protein samples were collected at 48 and 72 hours post‐transfection, respectively, for further detection.

### 2.3 Quantitative real‐time polymerase chain reaction (qRT‐PCR)

Total RNA was extracted from oesophageal cancer cells using TRIzol reagent (Life Technology). Next, cDNA was obtained from 2 μg of total RNA using a reverse transcription kit (TaKaRa, Tokyo, Japan). qRT‐PCR analyses of the expression of the *FOXM1a*,* FOXM1b*,* FOXM1c*,* FOXM1d* and *IRF1* genes were performed on an ABI Prism 7900 System with SYBR Premix Ex Taq II (TaKaRa). The primers were designed against the region that locates exclusively in each isoforms. And the specificity and amplification efficiency were verified previously.^14^ All primers are listed in Table S1. The data were analysed using QuantStudio^TM^ Real‐Time PCR software, and the relative expression was analysed using the 2^−ΔΔCt^ method. Three separate experiments were performed.

### 2.4 Preparation of a monoclonal antibody directed against the FOXM1 exon Va‐encoding sequence

A peptide identical to the FOXM1a/c exon Va‐encoding sequence (HWTQGLHNCPSTWN) was synthesized and then conjugated with keyhole limpet haemocyanin (KLH) as the immunogen. Then, the KLH‐conjugate protein was immunized in Balb/C mice to generate monoclonal antibody (McAb) according to a standard protocol. The specificity of this McAb, termed Va, was verified by Western blotting (Figure S1B).

### 2.5 Western blot

Cancer cells were harvested and lysed with cell lysis buffer (Sigma, St. Louis, MO) containing protease inhibitor cocktail and phosphatase inhibitor A and B (Selleck Chemicals, Houston, TX). The Western blot process was conducted according to the standard protocol, and the blots were visualized by an enhanced chemiluminescence (ECL) system. The antibodies against the following proteins were used in the immunoblotting assay: IRF1 (ET1602028; HuaAn Biotechnology, Hangzhou, China), MMP2 (ER40806; HuaAn Biotechnology), MMP9 (ET1704‐69; HuaAn Biotechnology), E‐cadherin (ab40772; Abcam, Cambridge, MA), vimentin (#133260; Cell Signaling Technology, Danvers, MA), snail (#3879; Cell Signaling Technology) and β‐actin (Sc47778; Santa Cruz, Santa Cruz, CA).

### 2.6 Immunohistochemistry (IHC) assay

Cancer tissues from 120 paraffin‐embedded ESCC patients were obtained from the tissue bank at Fudan University Shanghai Cancer Center, and utilization of samples was approved by the Ethics Committee at Fudan University Shanghai Cancer Center. The ESCC paraffin‐embedded tissues were cut into 5‐μm‐thick slices. The in situ expression of FOXM1c and IRF1 was detected by IHC staining using an anti‐Va antibody and anti‐IRF1 (ET1602028; HuaAn Biotechnology) antibody. Briefly, slices were deparaffinized in xylol, heated for antigen retrieval using 10 mmol/L sodium citrate (pH 6.0), treated with 3% hydrogen peroxide to inhibit endogenous peroxidase activity and blocked using 1% BSA/PBS. Slices were put in a wet box and incubated with anti‐Va and anti‐IRF1 antibody at 4°C overnight. Reactions were developed using GTvision^TM^ III (GK500710; Gene Technology, Shanghai, China) and counterstained with 10% haematoxylin. Finally, slices were dehydrated and mounted with resinene. The staining index (0‐12) was defined as the staining intensity (negative (0); weak (1); moderate (2); strong (3)) multiplied by the proportion of positive staining (0%‐25% (1); 25%‐50% (2); 50%‐75% (3); 75%‐100% (4)). The staining results were scored by two experienced pathologists blinded to the clinical data.

### 2.7 Construction of reporter plasmids

A 958 bp sequence from −820 to +138 bp of IRF1 (NM_002198.2) relative to the transcriptional start site was subcloned into the *Kpn*I and *Xho*I sites of the pGL3‐basic vector (Promega), using the following primers: forward‐*Kpn*I: 5'‐CGGGTACCCGACCTTGAAAACTACTCAGC‐3' and reverse‐*Xho*I: 5'‐CCTCTCGAGAAGAGGGAAGAAGGCAGAG‐3'. Four truncated vectors were also established based on potential FOXM1 binding sites predicted in the website https://www.genomatix.de/URLHASH;. All reporter plasmid constructs were verified by sequencing.

### 2.8 In vitro migration and invasion assay

For transwell assay, cancer cells were digested and counted after transient or stable transfection and were seeded with 5 × 10^4^ cells with 200 μL serum‐free RPMI 1640 medium were seeded in the upper well (8 μm pore; Corning, Inc., Corning, NY, USA) (serum‐free medium) with or without a matrigel‐coated membrane for the invasion or migration assays, respectively, following addition of complete RPMI 1640 medium with 10% FBS in the lower well. After culturing for 24, 48 or 72 hours as indicated in the figure legends of Figures 2 and 3, images of three random fields per well were obtained (200×) and used for quantification.

For wound healing assays, Eca109 cells were transfected with FOXM1c‐, IRF1‐specific siRNA, or scramble siRNA when the confluency reached 80%. Twenty‐four hours after transfection, cells were digested and plated into six‐well plates with 90% confluency. The next day a wound was created by manually scratching the cell monolayer with a 200 μL pipette tip. The culture medium was discarded, and the plates were washed twice with 0.1 M PBS to remove floating cells and cell debris. The cells were then incubated in RPMI‐1640 medium supplemented with 2% FBS. Cell migration into the wound was observed at three time points (0, 24 and 48 hours) in three randomly selected microscopic fields for each condition and time point. The images were captured using phase microscope and analysed with Image J software (NIH, Bethesda, MD, USA) by measuring the wound healing area. The rate of wound healing (scratch closure) = [(wound area at 0 hour − wound area at 24 or 48 hours)/wound area in 0 hour] ×100%.

### 2.9 Dual luciferase assay

For analysis of the effect of FOXM1c on regulating IRF1 transcription, we employed dual luciferase reporter assays as described previously.^29^ In brief, 293T cells were transiently transfected with the different pGL3‐IRF1 plasmids together with FOXM1c‐expressing or FOXM1c shRNA plasmid. The IRF1 promoter activity was normalized via co‐transfection with a Renilla luciferase reporter gene. The luciferase activity was quantified using a dual luciferase assay kit (Promega) 48 hours after transfection.

### 2.10 Chromatin immunoprecipitation (ChIP) assay

ChIP assays were used to identify the physical binding of FOXM1c to the IRF1 promoter. Oesophageal cancer cells (2 × 10^6^) were prepared for ChIP assays using a ChIP assay kit (Merck Millipore, Billerica, MA) according to the manufacturer's protocol. The resulting immunoprecipitated DNA specimens were analysed using three ChIP primers to amplify three regions of the IRF1 promoter; the PCR products were 198, 220 and 194 bp. The ChIP primers are as follows: 5'‐GATTTCCCCTGGTCCAGCA‐3' (forward) and 5’‐GAATCTCCCGACTGGCAGC‐3’ (reverse). The PCR products were resolved electrophoretically on a 2% agarose gel and visualized using ethidium bromide staining.

### 2.11 Statistical analysis

Statistical evaluation was conducted with SPSS 22.0 (SPSS Inc., Chicago, IL). The chi‐square test was used to analyse the relationship between clinicopathological parameters and the expression of CD59. The 5‐year overall survival (OS) and disease‐free survival (DFS) were calculated by the Kaplan‐Meier method, and differences in variables were compared using log‐rank tests. The significance of the in vitro and in vivo data was determined using Student's *t* test (two‐tailed). All data are shown as the mean ± SD Experiments were repeated at least three times. *P* values less than 0.05 were considered significant.

## 3 RESULTS

### 3.1 FOXM1c was the predominant isoform in ESCC cells

**Figure 1:**
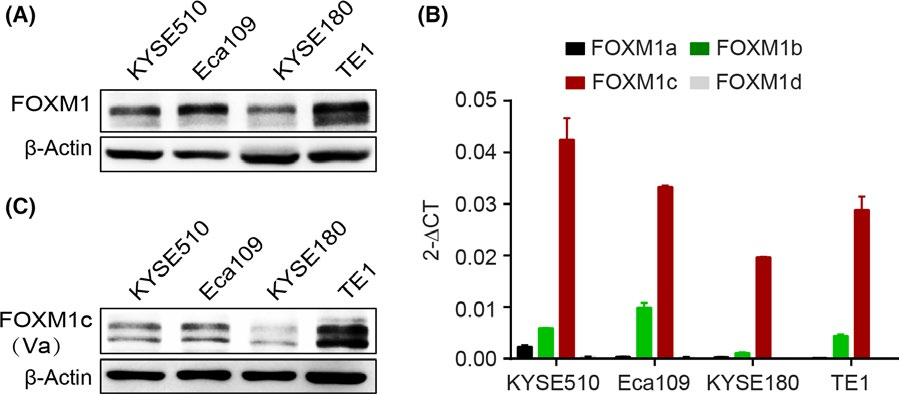
FOXM1c dominated among the four isoforms of FOXM1. (A) Total FOXM1 expression levels determined by Western blotting. (B) The mRNA level of each FOXM1 isoform measured by quantitative RT‐PCR. (C) FOXM1c expression detected by Western blotting. Va, the mouse monoclonal antibody against the exon Va‐encoded peptide

Although FOXM1 was highly expressed in oesophageal cancer and correlated with poor prognosis,^25^, ^30^, ^31^ the major isoform involved remains unknown. Before identifying the abundancy of each FOXM1 isoform in oesophageal cancer, we employed four ESCC cell lines to detect the expression levels of whole FOXM1 by Western blot. The result showed that FOXM1 was profusely expressed in a comparable level among these cells (Figure 1A). Due to the alternative splicing of exons Va and VIIa,^13^, ^14^ it is difficult to individually separate the distinct four isoforms of FOXM1 by Western blotting. Thus we used quantitative RT‐PCR to measure the mRNA levels of each FOXM1 isoform with specific primers.^14^ We observed that the FOXM1c isoform was uniformly expressed at a much higher level than the other three isoforms in all four cell lines (Figure 1B and Figure S1A), indicating the potential importance of FOXM1c in oesophageal cancer progression. The mRNA level of another isoform, FOXM1b, followed that of FOXM1c but was at a much lower level, whereas the other two isoforms, FOXM1a and FOXM1d, were undetectable at the mRNA level (Figure 1B).

To further verify the expression of FOXM1c at the protein level, we generated a mouse monoclonal antibody (McAb) directly against the exon Va (thus named Va), which is only contained in FOXM1a and FOXM1c.^32^ Considering that FOXM1a was negligibly expressed in ESCC (Figure 1B) and in pancreatic and colorectal cancers as described previously,^14^, ^33^ we argued that this McAb Va mainly recognized FOXM1c at least at the above cancer types. Next, we verified the specificity of the McAb Va. As shown in Figure S1B, this McAb against exon Va specifically recognized the ectopic expression of FOXM1a and FOXM1c but not of FOXM1b and FOXM1d in KYSE510 cells by Western blotting. Further, using this McAb, we found that the pattern of FOXM1c expression in four ESCC cells was consistent with FOXM1 expressed at similar levels (Figure 1C). Therefore, we concluded that FOXM1c was the predominant isoform in ESCC cells.

### 3.2 Genetic alteration of FOXM1c expression level affected ESCC cell metastasis

**Figure 2:**
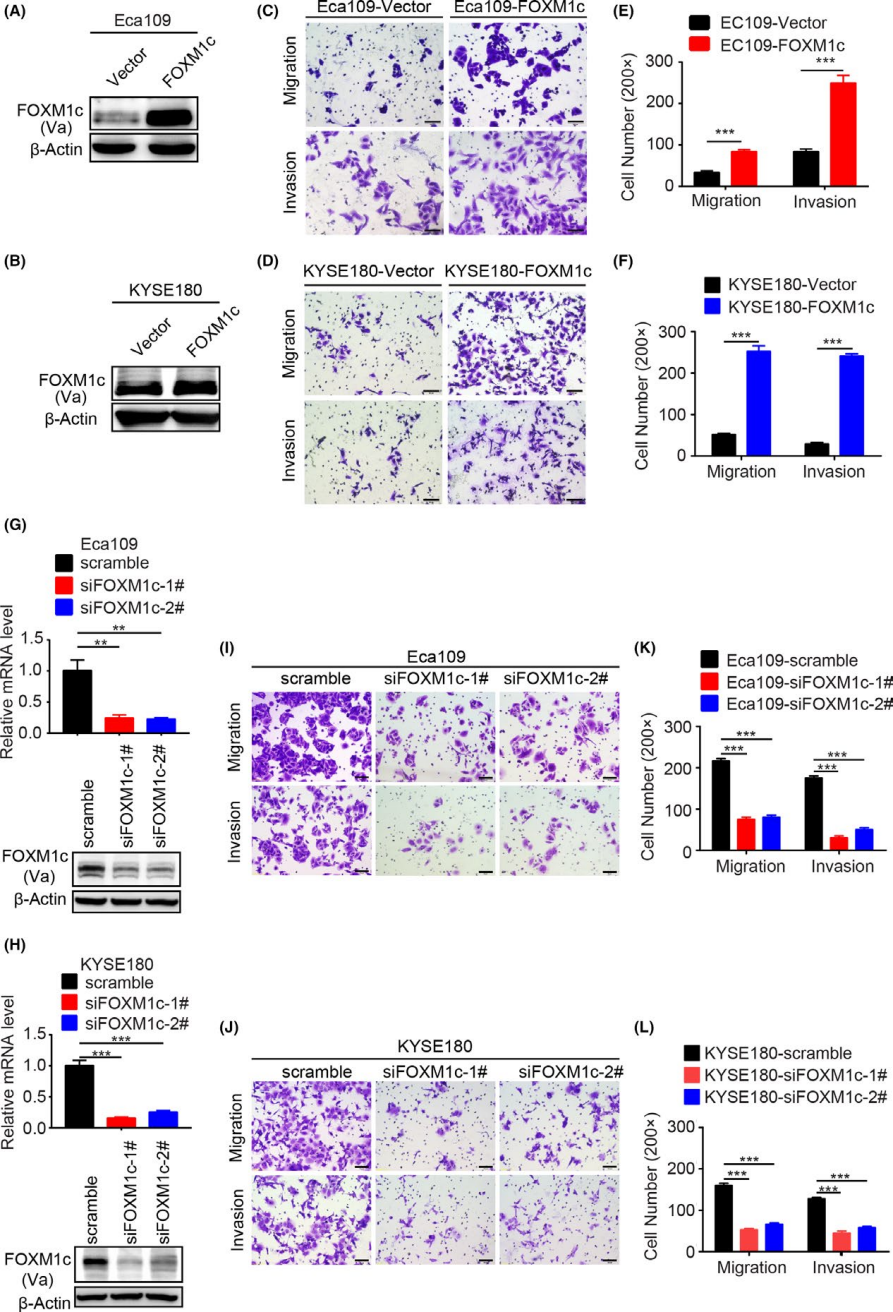
Genetic alteration of FOXM1c expression levels affected oesophageal cancer invasion and migration. (A,B) Verification of ectopic FOXM1c expression in Eca109 (A) and KYSE180 (B) cells. (C‐F) Ectopic FOXM1c expression significantly enhanced migration and invasion of Eca109 (C and E) and KYSE180 (D and F) cells. The migration and invasion activities were detected at 24 h or 48 h after plating, respectively. Quantitative results in E and F. (G‐H) Verification of FOXM1c insufficiency by the specific siRNA in Eca109 (G) and KYSE180 (H) cells. (I‐L) FOXM1c insufficiency significantly suppressed migration and invasion of Eca109 (I, K) and KYSE180 (J and L) cells. The migration and invasion activities were detected at 48 h or 72 h after plating, respectively. Quantitative results in K and L. Data represent the mean ± SD; n = 3; ***P < 0.001; and analysis with Student's *t* test (unpaired, two‐tailed)

Given the evidence that FOXM1 coincides with metastasis of breast cancer, pancreatic cancer and prostate cancer,^28^, ^34^, ^35^ we next ectopically overexpressed FOXM1c in three oesophageal cancer cell lines, Eca109, KYSE180 (Figure 2A,B) and KYSE510 (Figure S2A). Then, migration and invasion were assessed with transwell assays. Compared to the vector control group, the ectopic FOXM1c expression groups showed dramatically promoted migration and invasion of all oesophageal cells (Figure 2C,D and Figure S2B). The related quantitative results are shown in Figure 2E,F and Figure S2C.

To further confirm the role of FOXM1c in promoting metastasis of oesophageal cancer, we performed siRNA‐based knockdown of *FOXM1c* in the above three cell lines. We observed dramatically decreased expression of FOXM1c at both the mRNA and protein levels after *FOXM1c* knockdown (Figure 2G,H and Figure S2D). Compared to scramble siRNA‐transfected cells, all the cells transfected with siFOXM1c‐1# and siFOXM1c‐2# consistently showed a significant decrease in invasion and migration detected by transwell assays and wound healing assays (Figure 2I,J, Figures S2E and S3A). The associated quantitative results are shown in Figure 2K,L and Figures S2F and S3B. Taken together, these results indicated that genetic alteration of FOXM1c expression strongly affected the invasion and migration of oesophageal cancer cells; thus, FOXM1c may play a critical role in oesophageal cancer metastasis.

### 3.3 IRF1 mediated FOXM1c‐induced cell migration and invasion via MMP2/9

**Figure 3:**
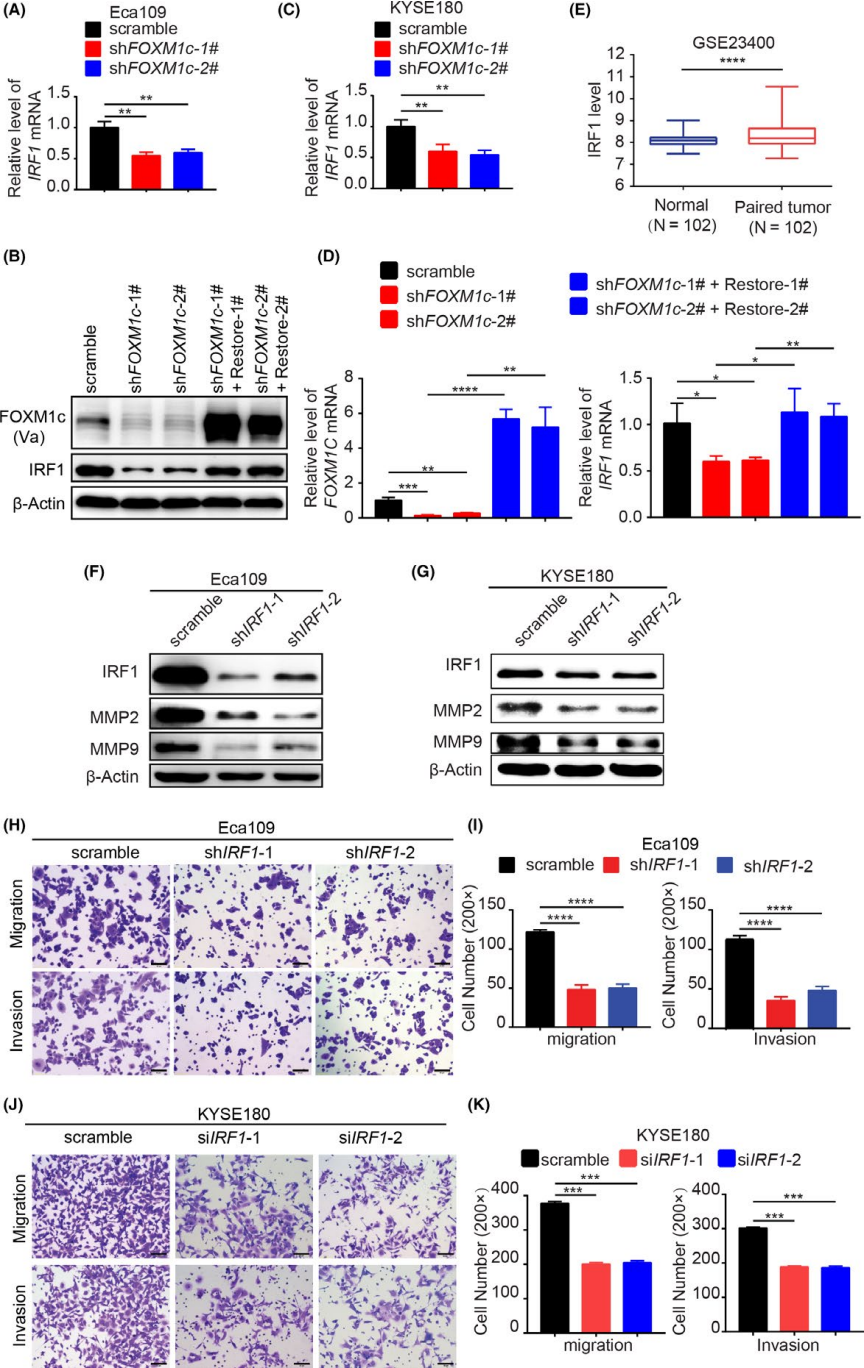
IRF1 mediated FOXM1c‐induced cell migration and invasion via MMP2/9. (A‐D) Knockdown of FOXM1c reduced the expression of IRF1. FOXM1c insufficiency suppressed IRF1 expression in Eca109 (A and B) and KYSE180 (C) cells. Restoring FOXM1c expression in the Eca109‐sh*FOXM1c*‐1# and ‐sh*FOXM1c*‐2# cells rescued the expression of IRF1 in mRNA (D) and protein (B) level. (E) The relevance of IRF1 expression between oesophageal cancer and paired normal tissues with GSE analysis (n = 102). (F‐G) shRNAs specifically targeting IRF1 resulted in the downregulation of IRF1 and consequently MMP2 and MMP9 in Eca109 (F) and KYSE180 (G) cells. (H‐K) IRF1 insufficiency suppressed migration and invasion of Eca109 (H and I) and KYSE180 (J and K) cells. The migration and invasion activities were detected at 48 h or 72 h after plating, respectively. Quantitative results in I and K. Data represent the mean ± SD; n = 3; *P < 0.05; **P < 0.01; ***P < 0.001; ****P < 0.0001; and analysis with Student's *t* test (unpaired, two‐tailed)

To identify the downstream targets that are potentially regulated by FOXM1c and simultaneously contribute to oesophageal cancer metastasis, we next tested 15 previously reported genes that are regulated by FOXM1 and highly associated with cancer metastasis (Figure S4).^23^, ^36^, ^37^, ^38^, ^39^, ^40^ To identify which gene is responsible for FOXM1c regulation of ESCC cell metastasis, we assessed the alterations of mRNA levels of 15 genes by RT‐PCR after knocking down FOXM1c expression with specific siRNA. The results showed that only the IRF1 mRNA was consistently decreased by FOXM1c insufficiency in all three tested ESCC cell lines (Figure S4). The FOXM1c knockdown induced by shRNA further confirmed that FOXM1c insufficiency reduced IRF1 transcription in Eca109 (Figure 3A), KYSE180 (Figure 3B) and TE1 (Figure S5) cells. We further enforced the expression of FOXM1c in Eca109‐sh*FOXM1c*‐1# and Eca109‐sh*FOXM1c*‐2# stable cell lines by transiently transfecting FOXM1c‐expressing plasmids with synonymous mutations in the wobble positions of codons in the shRNA target region. The results showed that IRF1 expression was consequently upregulated in both mRNA and protein levels (Figure 3B,D). In addition, based on the GEO public database analysis, we found that IRF1 expression in oesophageal cancer was significantly higher than that in paired normal tissues, indicating a positive correlation between IRF1 and oesophageal cancer progression (GSE23400) (Figure 3E). Therefore, we chose IRF1 for further investigation.

Furthermore, we silenced IRF1 with shRNA to identify the subsequent alterations of MMP2/9 and the effects on cell migration and invasion. The results demonstrated that both MMP2 and MMP9 were substantially downregulated in Eca109 and KYSE180 cells (Figure 3F,G). Accordingly, the cell migration and invasion capacities were strongly suppressed (Figure 3H,J, the quantitative results shown in Figure 3I,K). Knockdown of IRF1 with specific siRNAs also impaired the wound healing abilities (Figure S3C,D). Therefore, these findings suggest that FOXM1c promoted oesophageal cancer metastasis, at least in part, by regulating the IRF1‐MMP2/9 signalling axis.

### 3.4 FOXM1c regulated IRF1 transcription

**Figure 4:**
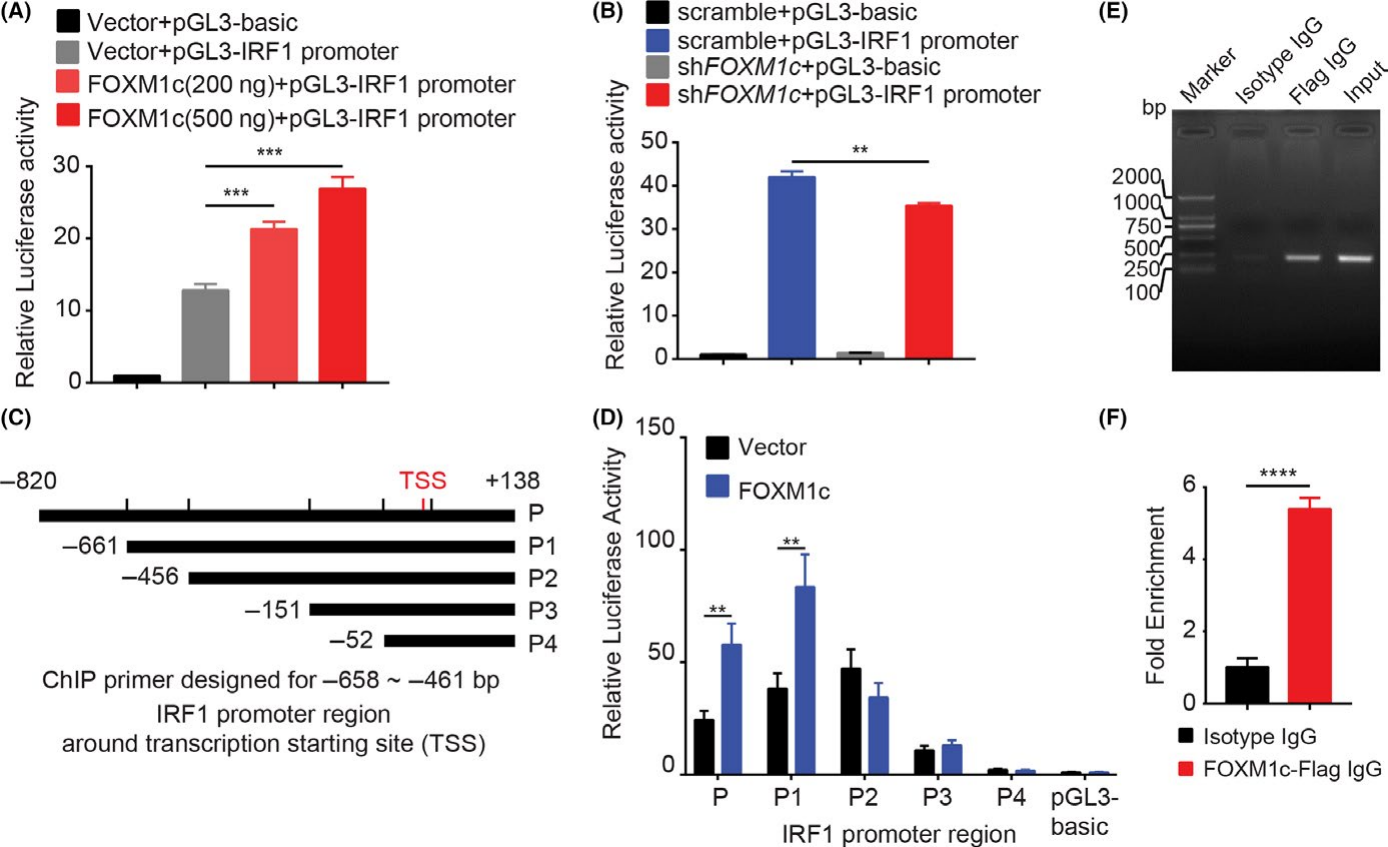
FOXM1c is a transcription factor of IRF1. (A) IRF1 promoter activity, represented by relative luciferase activity, was enhanced by ectopic FOXM1c expression in a dose‐dependent manner in 293T cells. (B) FOXM1c insufficiency significantly suppressed IRF1 promoter activity in 293T cells. (C) The truncated IRF1 promoter regions around the TSS were cloned into the pGL3 plasmid. (D) Ectopic FOXM1c expression strongly enhanced the promoter activity of the P and P1 but not the P2, P3 and P4 regions. In addition, when only transfecting control vector, the promoter regions of P, P1, P2 and P3 displayed a significantly increased relative luciferase activity compared with P4 and pGL3‐basic plasmid, indicating multiple promoter regions located in a P fragment other than the FOXM1c binding site. (E‐F) ChIP assays performed in 293T cells. A specific anti‐Flag antibody for ectopically expressed Flag‐FOXM1c, but not isotype IgG, captured the fragment containing the FOXM1c response element in the IRF1 promoter region, which was amplified by specific primers using PCR (E). The quantitative data in F. Data represent the mean ± SD; n = 3; **P < 0.0; ***P < 0.001; ****P < 0.0001; and analysis with Student's *t* test (unpaired, two‐tailed)

To further reveal the mechanism of IRF1 transcription regulated by FOXM1c, we employed dual luciferase reporter assays by cloning the IRF1 promoter region (−820 bp to +138 bp) into the pGL3 vector to generate the reporter pGL3 plasmid. Next, this plasmid was co‐transfected into 293T cells with the pRL inter‐control plasmid and different doses of the FOXM1c‐expressing plasmid. The results showed that the transcriptional activity represented by the relative luciferase activity was gradually elevated by the FOXM1c plasmid in a dose‐dependent manner (Figure 4A). In contrast, there was a significant decrease in transcriptional activity in FOXM1c knockdown cells compared to control cells (Figure 4B). These results indicate that FOXM1c may be involved in IRF1 transcription. We also noted that the transcriptional activity of the IRF1 promoter remained at a high level in FOXM1‐insufficient cells, indicating that other transcription factors may also participate in IRF1 transcriptional regulation.

To further determine the FOXM1c binding sites in the IRF1 promoter region, we constructed a series of pGL3 plasmids containing 5' truncations of the IRF1 promoter with different lengths (Figure 4C). These plasmids were then co‐transfected into 293T cells with the FOXM1c‐expressing plasmid or empty vector. The results of relative luciferase activity showed that ectopic FOXM1c expression significantly elevated transcriptional activity of the plasmids containing the P and P1 but not the P2, P3 and P4 IRF1 promoter regions compared to the vector control (Figure 4D). These results suggest that the FOXM1c binding site was most likely located in the region of −661 to −456 bp upstream of the transcription starting site (TSS).

We next performed ChIP assays to further verify the physical binding of FOXM1c to the promoter region identified above. After 293T cells were transiently transfected with the Flag‐FOXM1c‐expressing plasmid, we found that an anti‐Flag antibody but not isotype IgG could effectively capture the binding site of FOXM1c (Figure 4E,F). Therefore, we concluded that FOXM1c effectively regulated IRF1 transcription by directly binding to the specific promoter region.

### 3.5 IRF1 was highly associated with FOXM1c and both were correlated with oesophageal cancer progression

**Figure 5:**
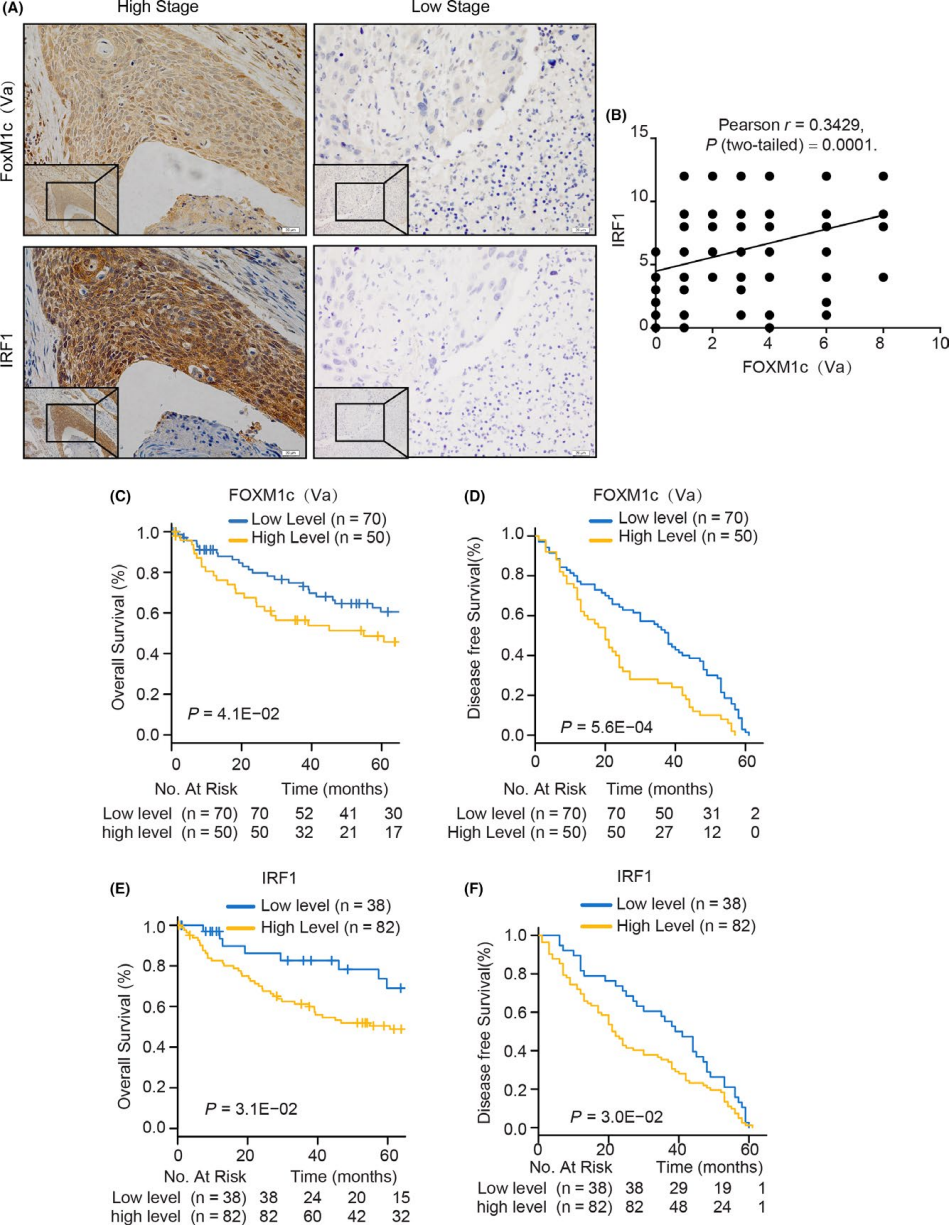
The association and clinical relevance of FOXM1c and IRF1 in 120 human oesophageal cancer tissues. (A) Representative images of FOXM1c and IRF1 staining in oesophageal cancer tissues with high and low stages. Scale bar, 20 μm. (B) The association between FOXM1c and IRF1 expression levels calculated by Pearson analysis. (C‐F) Kaplan‐Meier survival curves of OS (C and E) and DFS (D and F) based on FOXM1c (C and D) and IRF1 (E and F) expression levels in all cancer tissues. OS, overall survival; DFS, disease‐free survival

To investigate the relationship between the expression levels of FOXM1c and IRF1 in clinical specimens, we collected 120 oesophageal cancer samples (Table 1), which were classified as high or low stage based on their TNM classifications. The expression levels of FOXM1c and IRF1 were determined by IHC with the specific antibodies. We found that IRF1 expression was highly associated with FOXM1c. The representative images are shown in Figure 5A, in which both FOXM1c and IRF1 were strongly expressed in the samples classified as high stage, while slightly expressed or undetectable in the low‐stage samples (Figure 5A). The statistical analysis showed a significant positive correlation between FOXM1c and IRF1 (Figure 5B). Moreover, these oesophageal cancer patients were followed up from the year 2009 to 2012 to determine the OS and DFS. Patients with high FOXM1c and IRF1 expression were found to have shorter OS and DFS (Figure 5C‐F) and a high tumour stage (Table 1). Together, these results indicate that both FOXM1c and IRF1 were independent prognostic indicators and might be potential drug targets for oesophageal cancer.

**Table 1:** The association of the expression of FOXM1c and IRF1 with the clinicopathological features from ESCC patients (n = 120)

	FOXM1c (Va)			IRF1		
	Low level	High level	*P*	Low level	High level	*P*
Age						
≤60	38	14	0.689	17	35	0.849
>60	47	21		21	47	
Sex						
Male	75	23	0.587	36	62	0.807
Female	15	7		7	15	
Stage						
I	18	5	0.001^b^	10	13	0.008^a^
II	38	20		23	35	
III	14	25		5	34	
T stage						
1‐2	52	19	<0.001^b^	23	48	0.845
3‐4	18	31		15	34	
N stage						
0	28		0.35	19	25	0.013^a^
1	31	21		17	35	
2‐3	11	13		2	22	
Grade						
High	4	4	0.911	4	4	0.04^a^
Moderate	44	31		28	47	
Low	22	15		6	31	
Chemotherapy						
No	39	27	0.548	25	41	0.119
Yes	31	23		13	41	
Radiotherapy						
No	52	32	0.312	33	51	0.009^a^
Yes	18	18		5	31	
Total	85	35		38	82	

a *P* < 0.05;

b *P < *0.01.

## 4 DISCUSSION

FOXM1 produces four isoforms due to alternative splicing. FOXM1a function has been poorly characterized due to its extremely low expression, FOXM1b and FXOM1c mainly regulate oncogene transcription in the nucleus, and FOXM1d promotes cancer EMT and progression via interactions with ROCKs in the cytoplasm.^13^, ^14^ Although FOXM1b, FOXM1c and FOXM1d were investigated in certain cancer types,^14^, ^35^, ^41^ the distribution and function of these isoforms in oesophageal cancer remain unknown. In the present study, we found that the FOXM1c isoform dominated among the four FOXM1 isoforms in oesophageal cancer cells. Genetically altering FOXM1c expression strongly affected oesophageal cancer metastasis by regulating IRF1 transcription, thus resulting in a change in MMP2/9 expression. The close correlation between FOXM1c and IRF1 levels was further determined in 120 oesophageal cancer specimens. In addition, high expression levels of FOXM1c and IRF1 were also significantly associated with poor prognosis and advanced stage of oesophageal cancer. These findings highlight the role and reveal the mechanism of FOXM1c in promoting oesophageal cancer metastasis.

Various MMPs are required in multi‐step processes during tumour metastasis by degrading the extracellular matrix surrounding the tumour.^42^ Numerous evidence demonstrates that MMP gene expression is mainly regulated at the transcriptional level via a wide range of transcriptional factors including AP‐1, PEA3, Sp‐1, β‐catenin/Tcf‐4 and NF‐κB in a tissue/cell‐specific manner.^43^ Although MMP‐2/9 are both gelatinases, their promoter regions exhibit the different composition of *cis*‐elements, thus resulting in the different binding transcription factors. AP‐2 and TP53 regulate MMP‐2 transcription, while NF‐κB, PEA‐3 and AP‐1 regulate MMP‐9 transcription.^43^, ^44^, ^45^ Here, we interestingly found that the transcription factor of IRF1 simultaneously regulated both MMP‐2 and MMP‐9 in oesophageal cancer cells, possibly at the transcriptional level that requires future investigation, thus affecting cancer metastasis.

As a transcription factor, FOXM1 regulates many genes that are involved in different stages of cancer, including initiation, progression and metastasis.^13^, ^21^, ^32^ We screened a series of genes that are important for tumour metastasis and highly associated with FOXM1 expression. IRF1 was uniformly identified to be significantly downregulated by FOXM1c insufficiency in three oesophageal cancer cell lines. IRF1 is a transcription factor that regulates a number of IFN‐inducible genes in response to viral infection or interferon stimulation.^46^ The role of IRF1 in cancer progression is controversial depending on cancer types.^47^, ^48^ Our current study demonstrated that IRF1 was transcriptionally regulated by FOXM1c and was an important regulator for the oesophageal cancer cell invasion and migration via MMP2/9. We also found that there was a high correlation between FOXM1c and IRF1 in 120 oesophageal cancer specimens; more importantly, both FOXM1c and IRF1 were co‐overexpressed in oesophageal cancer patients in the advanced stage and with poor prognosis. Therefore, FOXM1c and IRF1 may be potential independent biomarkers for prediction of oesophageal cancer prognosis.

In summary, we determined that FOXM1c was the predominant isoform among the four isoforms of FOXM1 in oesophageal cancer. Moreover, we unveiled a novel mechanism of FOXM1c in regulating cancer invasion and migration, that is, FOXM1c transcriptionally regulated IRF1 by directly binding to its promoter region, and IRF1 further modulated MMP2/9 expression. Using clinical samples, we further demonstrated the close correlation between FOXM1c and IRF1 expression levels, and their expression levels were highly associated with oesophageal prognosis. These findings suggest the potential of FOXM1c and/or IRF1 as independent prognosis biomarkers or drug targets for oesophageal cancer.

## CONFLICT OF INTEREST

The authors declare no conflict of interest.
