# Supplementary material for: FOXM1c promotes oesophageal cancer metastasis by transcriptionally regulating IRF1 expression
Source: Cell Prolif. 2018 Nov 28;52(2):e12553. doi: 10.1111/cpr.12553 (PMC6496730; doi:10.1111/cpr.12553)

**Supplementary TABLE 1 qPCR primers**

| **gene** | **Primer(5'-3')** |
| --- | --- |
| **FOXM1a-F**  **FOXM1a-R** | GTCTCCACAATTGCCCGAG  CCAAAATCTCGCAGATCGC |
| **FOXM1b-F**  **FOXM1b-R** | GGTGTTTAAGCAGCAGAAAC  GCAGCACCTTGGGGGCAATGC |
| **FOXM1c-F**  **FOXM1c-R** | CCACTGGACCCAGGGTCTCC  GCAGCACCTTGGGGGCAATGC |
| **FOXM1d-F**  **FOXM1d-R** | CAGGTGTTTAAGCAGCAGA  GGTGATGGGTGTACCAAAAT |
| **IRF1-F**  **IRF1-R** | AAGGGGTGTGGCCTTTTTAGA  TGTCCCTGTTCACCCCAAAG |
| **ATCB-F**  **ATCB-R** | ACCGAGCGCGGCTACAG  CTTAATGTCACGCACGATTTCC |

**Supplementary Figure S1 The determination of the predominant FOXM1c isoform.**


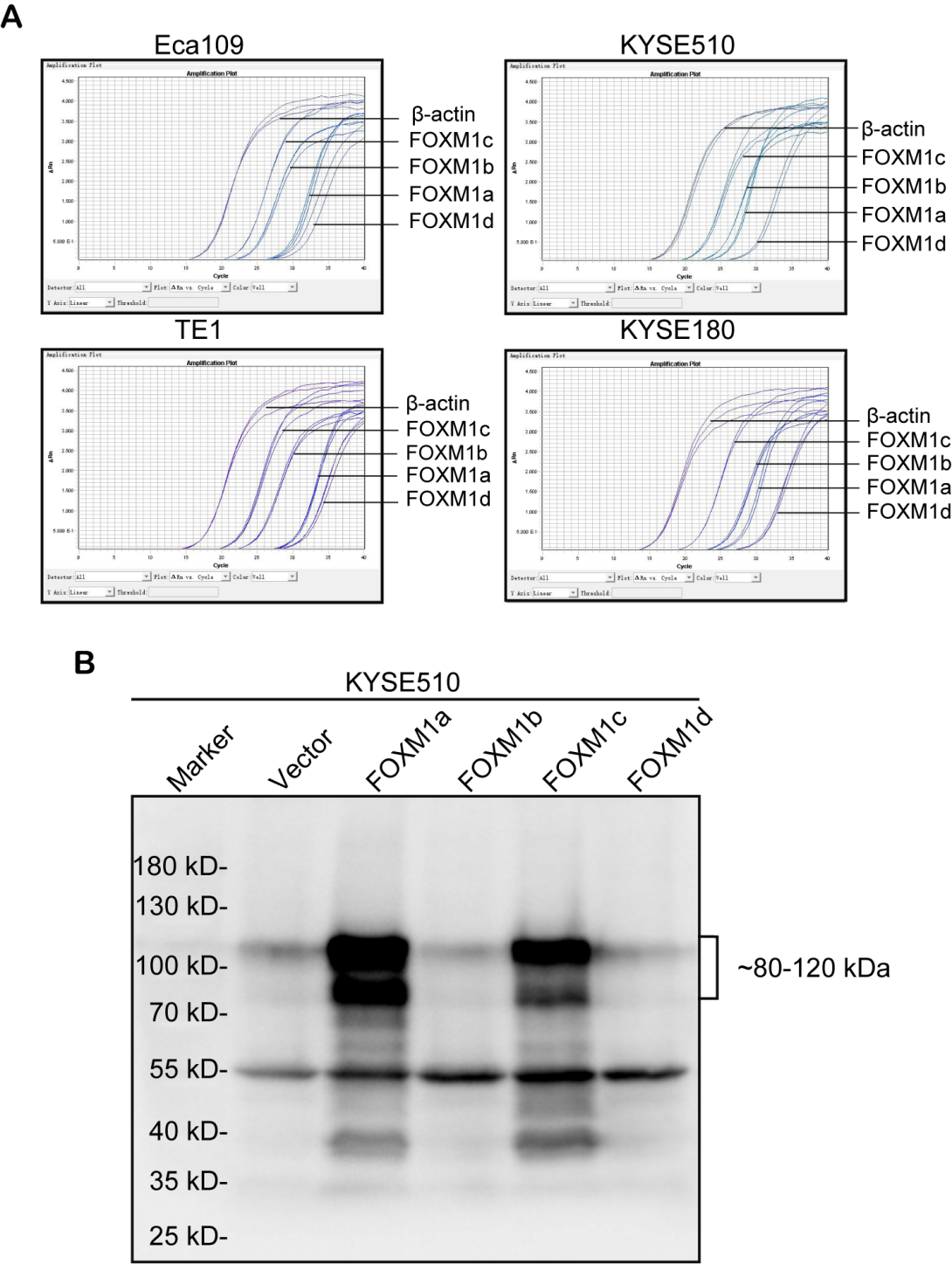


**Supplementary Figure S2 Genetic alteration of FOXM1c expression levels affected esophageal cancer invasion and migration in KYSE510 cells and TE1 cells.**


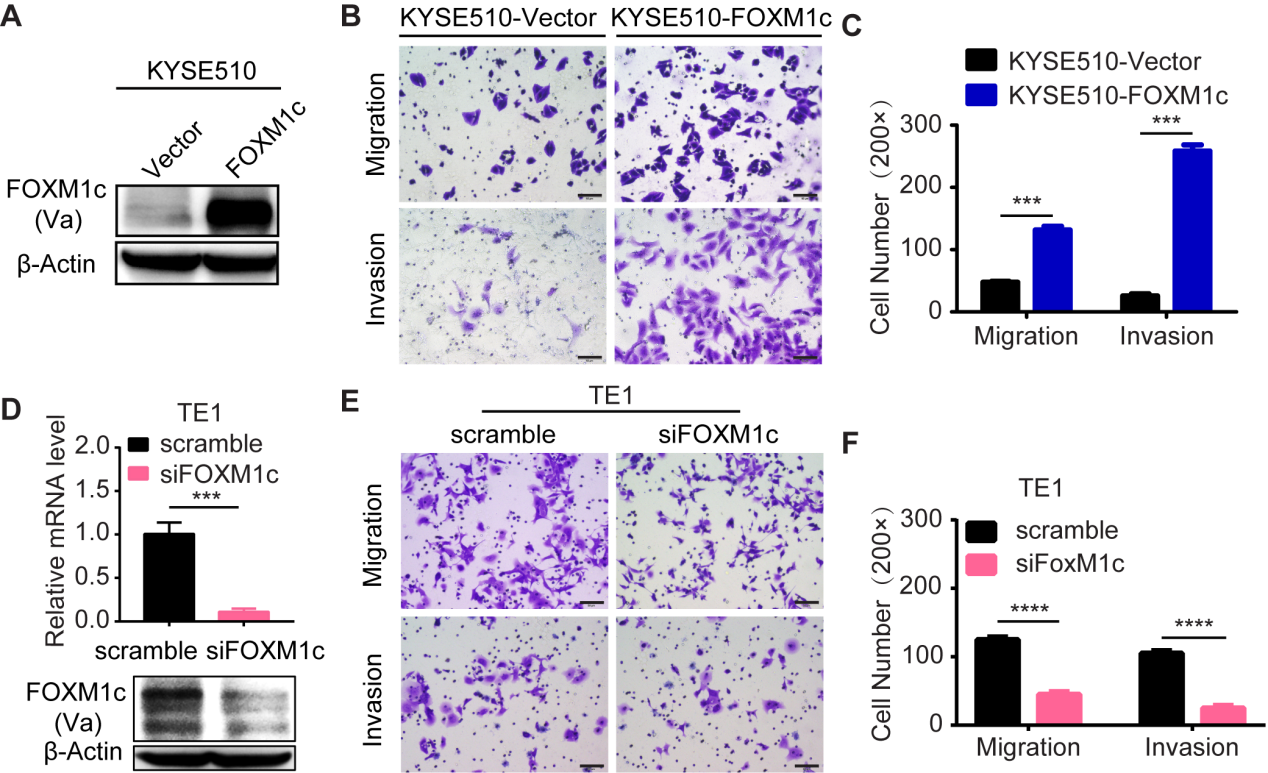


**Supplementary Figure S3 Insufficiency of FOXM1c and IRF1 by specific siRNAs suppressed the wound healing abilities of ESCC cells.**


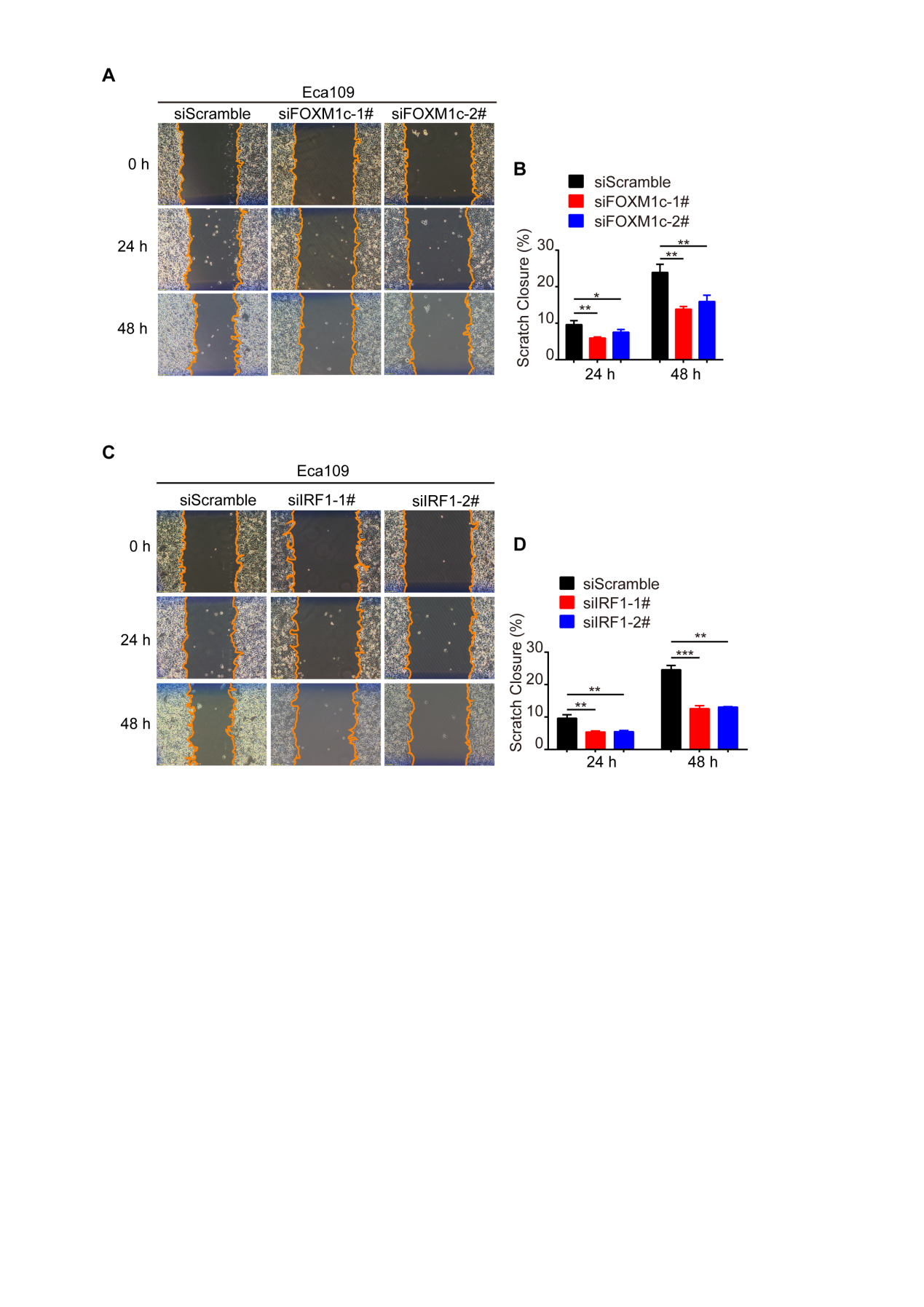


**Supplementary Figure S4 Identification of significantly affected genes by FOXM1c insufficiency.**


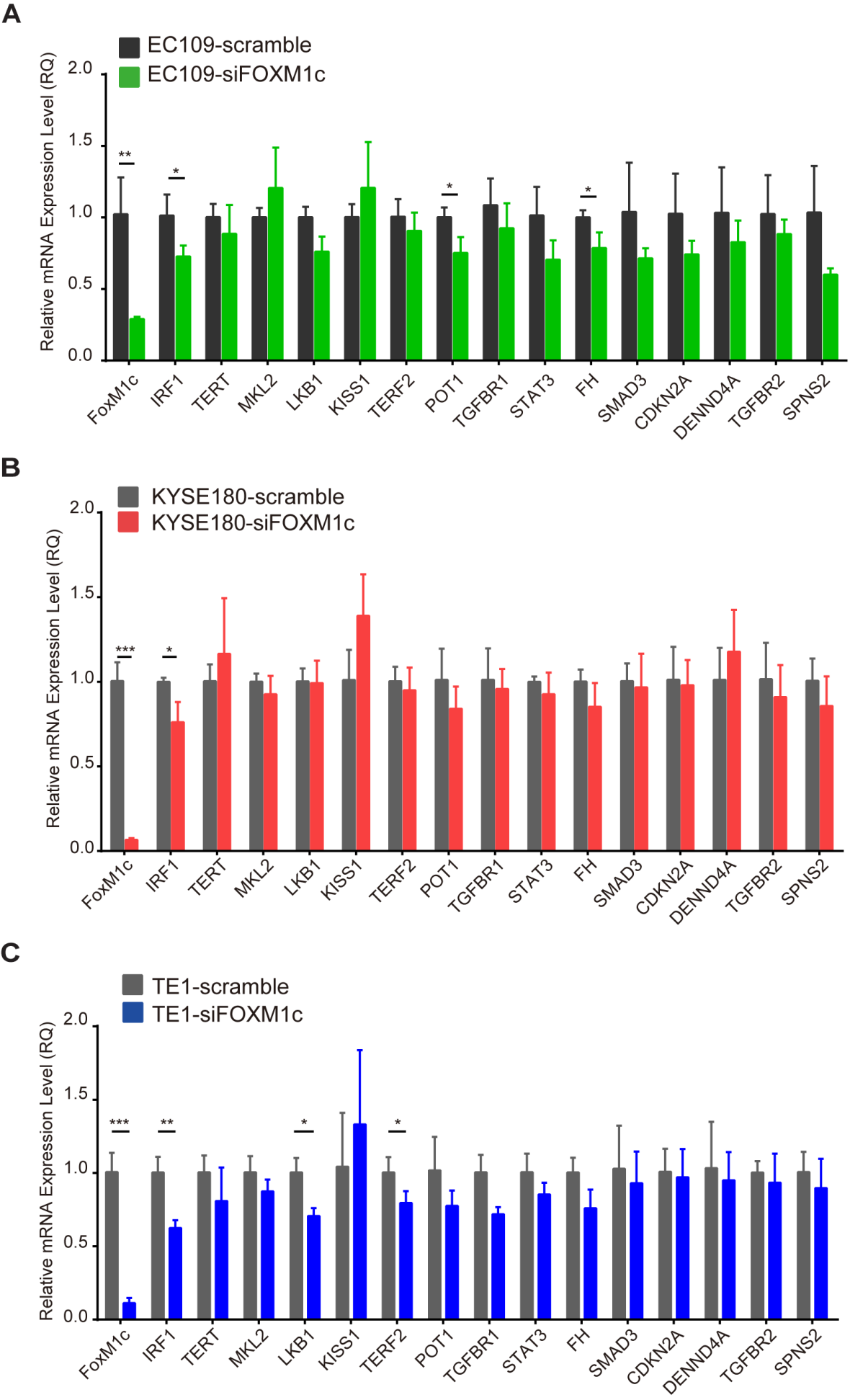


**Supplementary Figure S5 FOXM1c insufficiency reduced IRF1 expression in TE1 cells.**


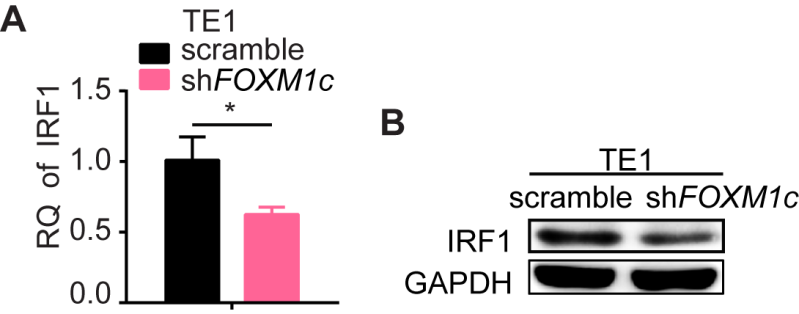

Supplement: Supplementary file 1 [file CPR-52-e12553-s001.docx]
